# A Residual Alignment and Dynamic Multi-Kernel Network for Infrared–Visible Pedestrian Detection

**DOI:** 10.3390/s26206667

**Published:** 2026-10-21

**Authors:** Shipeng Han, Chaowen Chai, Min Hu, Hao Lv

**Affiliations:** 1 School of Biomedical Engineering, Air Force Medical University, Xi’an 710032, China; shipenghan@fmmu.edu.cn (S.H.); ccw_0824@xauat.edu.cn (C.C.); hummmin@163.com (M.H.); 2 Shaanxi Provincial Key Laboratory of Bioelectromagnetic Detection and Intelligent Perception, Xi’an 710032, China; 3 School of Information and Control Engineering, Xi’an University of Architecture and Technology, Xi’an 710055, China

**Keywords:** infrared–visible, pedestrian detection, multi-kernel, residual alignment

## Abstract

To tackle feature redundancy, cross-modality misalignment, and scale sensitivity inherent in infrared–visible fusion-based pedestrian detection, this paper proposes a novel multimodal pedestrian detection network, termed EFDNet, with residual alignment and dynamic multi-kernel designs. Specifically, an enhanced multi-scale feature extraction (EMFE) module is developed to boost the discriminative capacity of fine-scale features. By embedding the efficient multi-scale attention (EMA) mechanism into a faster block architecture, this module strengthens edge and texture feature representation with marginal computational overhead. On this basis, a feature alignment restoration module (FARM) is constructed to suppress edge blurring and feature deviation caused by thermal radiation diffusion. Adopting a parallel residual-alignment-fusion paradigm with cross-modal dual-dimensional interaction, it achieves joint pixel-level and feature-level alignment across spatial and channel dimensions. Finally, a dynamic multi-kernel channel attention (DMCA) module is introduced for hierarchical feature refinement. Equipped with parallel multi-scale convolutions and a lightweight weight generation branch, this module optimizes feature characteristics from scale and channel perspectives, thus improving detection robustness for pedestrian targets of diverse scales. Extensive experiments on the KAIST dataset demonstrate that EFDNet achieves competitive L-AMR scores of 6.73%, 8.03%, and 4.11% on the overall, daytime, and nighttime subsets, respectively. Further evaluations on the FLIR and LLVIP datasets reveal that the proposed EFDNet attains mAP_50_, mAP_75_, and mAP_50–95_ of 82.8%, 36.6%, 42.0% and 97.3%, 79.0%, 68.2%, respectively, outperforming state-of-the-art methods on partial metrics and exhibiting competitive advantages.

## 1. Introduction

Pedestrian detection serves as a core task bridging low-level visual perception and high-level decision-making and planning. It plays a vital role in practical scenarios such as autonomous driving and all-weather security surveillance, and acts as an important support for building the safety-redundancy capability of autonomous driving systems. The accuracy of pedestrian detection directly determines the reliability of subsequent path planning and the effectiveness of collision-avoidance strategies. However, in complex real-world scenarios such as low-light and back-light conditions, single visible-light imaging suffers from a sharp drop in signal-to-noise ratio, which triggers a dramatic increase in miss-detection rate and severely limits system robustness [1]. Although infrared imaging compensates for insufficient illumination via thermal radiation sensing, it suffers from sparse texture information and low thermal contrast, which readily yields ambiguous target localization. Since visible and infrared cameras follow distinct physical imaging mechanisms, their information characteristics are inherently complementary. Accordingly, dual-spectral fusion-based pedestrian detection has emerged as a prominent research hotspot, as it boosts detection performance in complex environments by exploiting the complementarity of multimodal information [2,3].

Most existing mainstream fusion methods adopt a technical framework composed of a dual-stream backbone network, feature fusion module, and detection heads [4,5,6]. These methods still exhibit shortcomings in terms of fusion methods, timing, and objects. Early fusion strategies mostly adopt simple operations such as weighted concatenation or channel splicing to directly mix heterogeneous features with different physical mechanisms at the shallow layers of the network. Such methods assume that multi-spectral information can bring direct performance gains, while ignoring the essential differences that visible textures are mainly determined by high-frequency illumination components and infrared signals are dominated by low-frequency blackbody radiation. Forced fusion will trigger mutual interference between cross-modal features, resulting in redundant parameter computations and the attenuation of key structural features of pedestrians. Subsequent studies have introduced manual priors such as gating mechanisms [7] and attention weights [8] to optimize the fusion process. Nevertheless, existing studies generally adopt a fusion-first, selection-later processing paradigm, where redundant information is progressively filtered through subsequent network layers. Such a mechanism fails to fundamentally address the issues of parameter redundancy and feature confusion. In nighttime scenarios dominated by thermal noise, the gating branches are susceptible to intricate background disturbances, simultaneously increasing both miss rate and false positive rate. This phenomenon reveals the inherent coupling bottleneck of conventional fusion paradigms.

To further suppress complex background interference and strengthen the representation of key features, existing studies have introduced channel or spatial attention mechanisms into the fusion stage. These methods generally generate attention weights relying on fixed-size convolution kernels, which assume stable target scales and fail to match the drastic scale variations in pedestrians in real-world scenarios [9]. Fixed convolution kernels cannot effectively focus on small-scale distant targets and tend to over-smooth edge details when processing large-scale close-range targets. In addition, the attention weights remain fixed after training and cannot be dynamically adjusted according to input content during the inference phase. In the presence of thermal halo diffusion at night, fixed attention mechanisms may incorrectly enhance background thermal halo regions and obscure the boundary information of pedestrian targets. Some studies adopt multi-branch convolutions to expand the receptive field for multi-scale target adaptation. However, the static branch weights lack flexibility when scales and scene contents change simultaneously, which instead amplifies noise interference [10,11].

Inspired by the transformer architecture in natural language processing, recent efforts have attempted to apply it to cross-modal feature alignment. The global self-attention mechanism is utilized to calculate the similarity between visible and infrared features, alleviating the inherent differences in local convolutions when processing heterogeneous spectral information. Global modeling strengthens the long-range dependencies between features, yet it requires pairwise interaction of token vectors across the entire image, leading to a square-level increase in computational complexity with image resolution. Moreover, global smoothing operations tend to lose edge and texture details in low-level features and blur pedestrian contours [12].

Meanwhile, advances in dense prediction in the frequency domain offer a new perspective for understanding cross-modal fusion. Octave convolution [13] decomposes feature maps into high-frequency and low-frequency components, preserving edge information while reducing spatial redundancy. FcaNet [14] reformulates channel attention as a frequency-domain operation and demonstrates that channel reweighting is mathematically equivalent to adaptive spectral filtering. Frequency-aware feature fusion [15] further casts cross-scale and cross-branch fusion as adaptive high-low pass filtering and offset-based resampling, directly addressing the common problems of intra-target inconsistency and boundary shift in dense prediction tasks. These studies suggest that effective fusion should adaptively balance high-frequency details and low-frequency context, rather than treating all frequency components equally. Nevertheless, existing frequency-domain methods generally require explicit spectral decomposition or elaborately designed filter banks, which introduce extra computational overhead and may not be directly compatible with lightweight detection backbones. Motivated by these observations, this paper explores a lightweight scheme that implicitly achieves adaptive frequency balancing from the perspective of channel-wise and kernel-wise attention.

To address the aforementioned issues, this paper reconstructs the multi-spectral fusion detection framework from the perspectives of feature extraction efficiency, cross-modal alignment accuracy, and dynamic scale adaptability, and proposes an efficient fusion and detection network (EFDNet). The main contributions of this work are summarized as follows:

(1) A lightweight multi-scale feature extraction (EMFE) module is proposed to integrate into the CSPDarknet [16] backbone. The module introduces an EMA [17] and multiple cascaded faster blocks built with partial convolution (PConv) [18]. Benefiting from the sparse connection strategy based on split- concatenation operation and efficient PConv, the module precisely captures abrupt thermal radiation variations along object boundaries and improves the feature representation of low-level semantics.
(2) A feature alignment and restoration module (FARM) following the residual-alignment-fusion paradigm is devised. FARM utilizes depth-wise convolution (DConv) to extract modality-specific features. Within FARM, the embedded reference-based feature enrichment (RBFE) [19] module and burst feature attention (BFA) [20] collaboratively compute cross-modal correlation information across spatial and channel dimensions to realize synchronous alignment at both the feature and pixel levels. Supported by iterative residual feature compensation, the proposed module effectively mitigates edge blurring artifacts caused by thermal radiation dispersion.
(3) A dynamic multi-kernel channel attention (DMCA) module is proposed as a multi-scale extension of efficient channel attention (ECA) [21]. It adopts three parallel convolutions with different kernel sizes to model multi-scale channel dependencies and generates dynamic adaptive weights for channel feature fusion. This design achieves fine-grained channel recalibration and enhances the network’s adaptability to drastic pedestrian scale variations.

## 2. Methods

### 2.1. Structure of EFDNet

**Figure 1:**
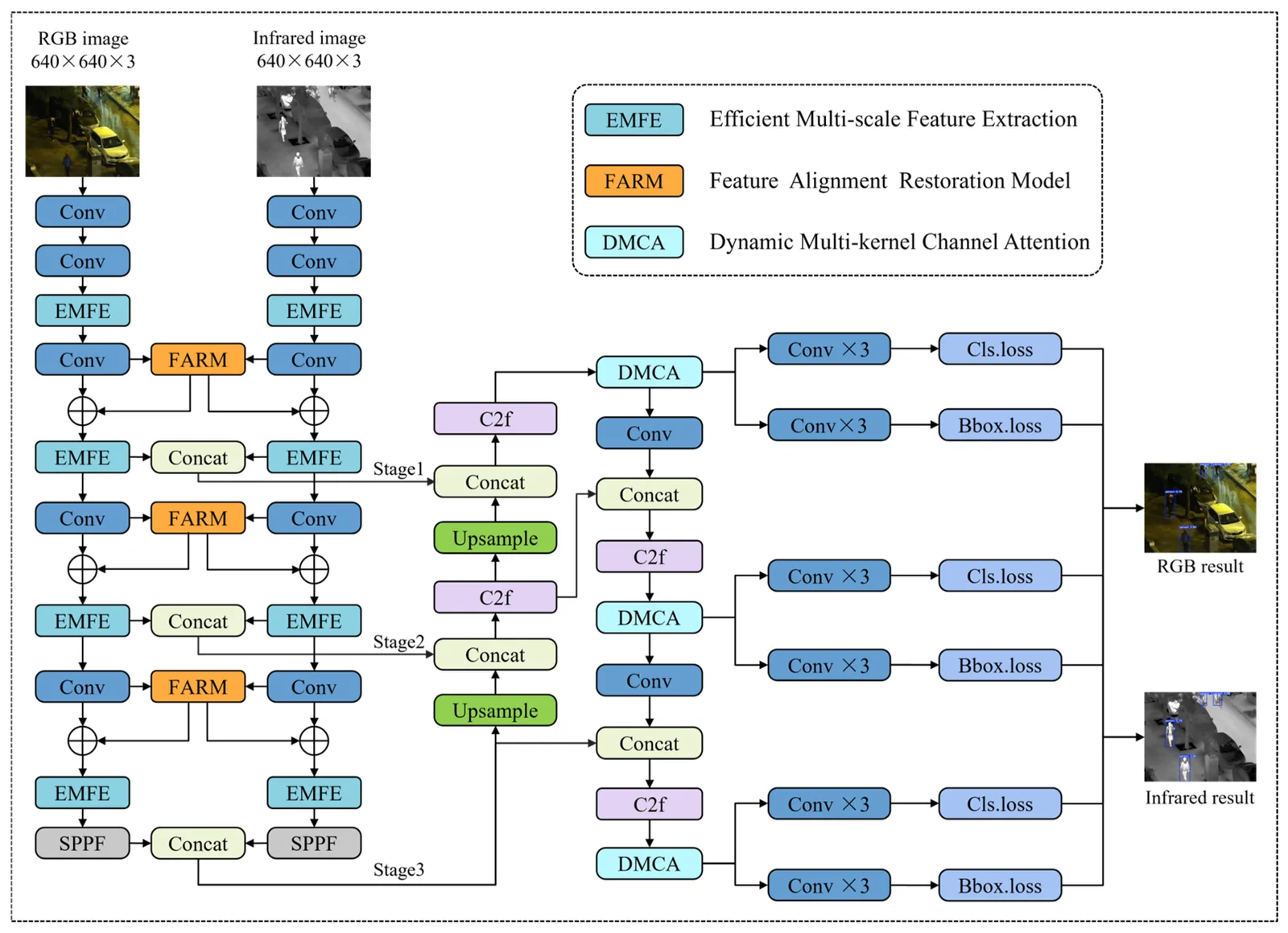
Overall architecture of the proposed EFDNet.

As illustrated in Figure 1, EFDNet is built upon the publicly available CSPDarknet backbone of two-stream YOLOv8n and adopts a dual-branch architecture to extract features from RGB and infrared images separately. At the input stage, raw infrared images are single-channel grayscale and are replicated three times along the channel dimension to form 3-channel inputs, satisfying the dimensional requirement of the CSPDarknet backbone pre-trained on RGB datasets. This operation introduces no additional information; the infrared branch solely learns thermal radiation features. After preprocessing, each pair of RGB and infrared images resized to a resolution of 640 × 640 × 3 is fed into two independent feature extraction branches.

For each single-modality branch, the input image first undergoes preliminary feature extraction through stacked standard convolutional layers. These convolutions capture low-level local texture, edge, and radiation cues, laying a solid foundation for subsequent cross-modal alignment and multimodal feature fusion. Following shallow convolutional processing, the extracted unimodal features are passed to the EMFE module. This lightweight feature extraction module performs intra-branch multi-scale feature mining and aggregation to enrich fine-grained feature representations and strengthen the discriminative capacity of unimodal feature maps.

To mitigate modal discrepancies between RGB and infrared images, FARM is deployed for cross-branch feature calibration and enhancement. Benefiting from spatial alignment operations, FARM effectively reduces feature distribution offsets induced by the inherent spectral differences between the two imaging modalities and unifies the spatial semantic consistency of cross-modal features. Meanwhile, residual connections embedded within FARM supplement the original feature information and further boost feature representation capacity, allowing the network to fully exploit the complementary information from both RGB and infrared images.

After hierarchical cross-modal feature alignment and concatenation-based fusion at backbone stages, multi-scale fused features are transmitted to the neck subnetwork. The neck adopts a top-down pyramid structure consisting of upsampling and concatenation operations to recover high-resolution spatial details and aggregate multi-level semantic features. C2f blocks are introduced to implement deep feature interaction and refine fused pyramid features. Afterwards, each scale branch of the feature pyramid is equipped with a DMCA module. This advanced attention mechanism adaptively assigns weights to feature maps processed by convolutional kernels of diverse sizes, achieving dynamic integration of multi-scale features. Such a flexible weight adjustment strategy improves the model’s adaptability to targets of varying sizes in complex cross-modal scenes.

In the final detection stage, three scale-specific detection heads are attached to the attention-enhanced pyramid features. Each detection head contains stacked convolutional layers and corresponding loss calculation branches. The classification branch predicts object categories under the supervision of classification loss, while the regression branch outputs bounding-box coordinates optimized via bounding-box loss. During inference, the network simultaneously outputs visualized pedestrian detection results overlaid on the original RGB and infrared images.

### 2.2. Efficient Multi-Scale Feature Extraction

**Figure 2:**
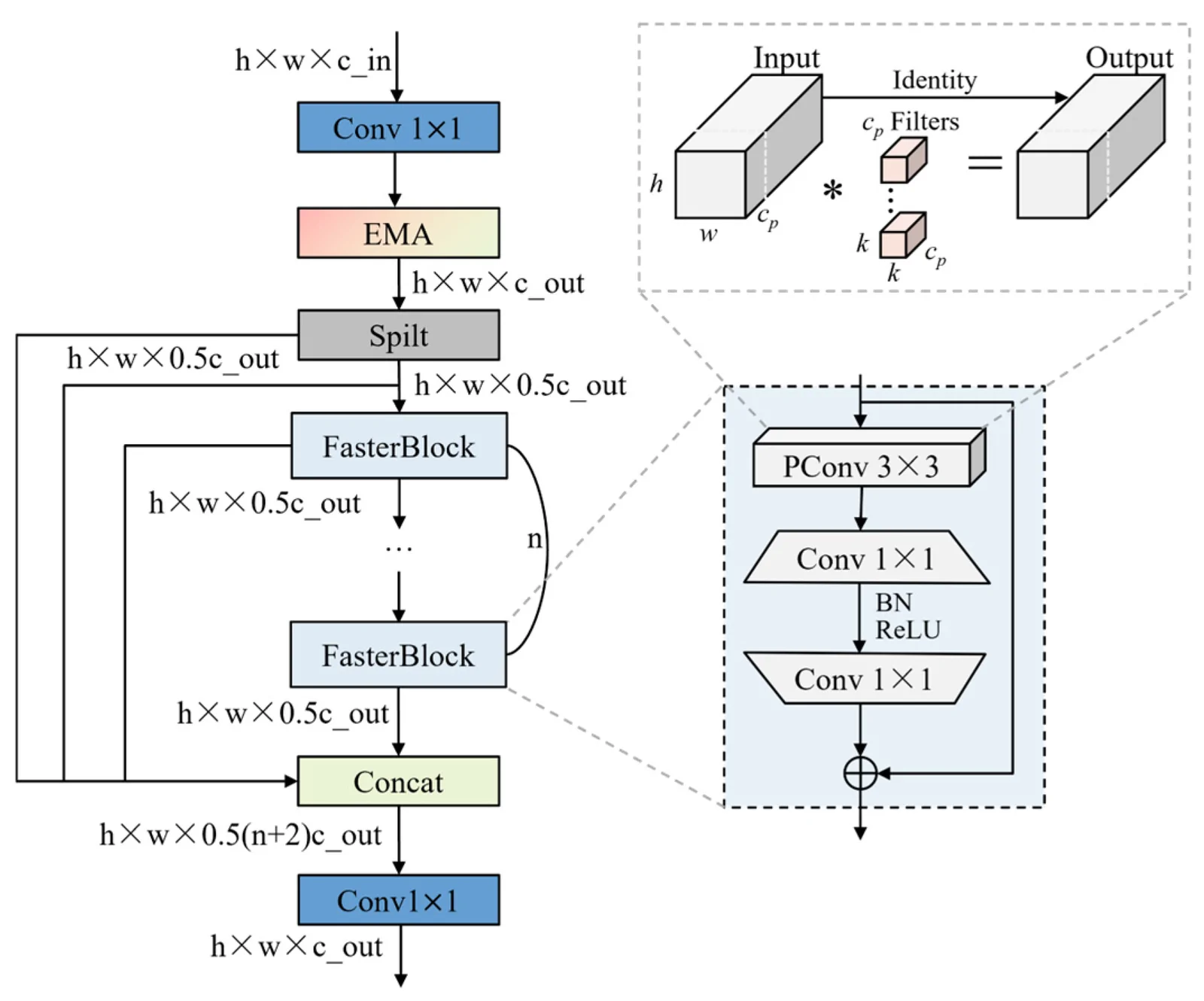
Structure of the proposed EMFE module.

To balance feature extraction efficiency and feature representation capability within RGB-infrared pedestrian detection, this work develops the EMFE module, which aims to excavate complementary discriminative cues from paired multimodal inputs. The overall structure of EMFE is visualized in Figure 2.

**Figure 3:**
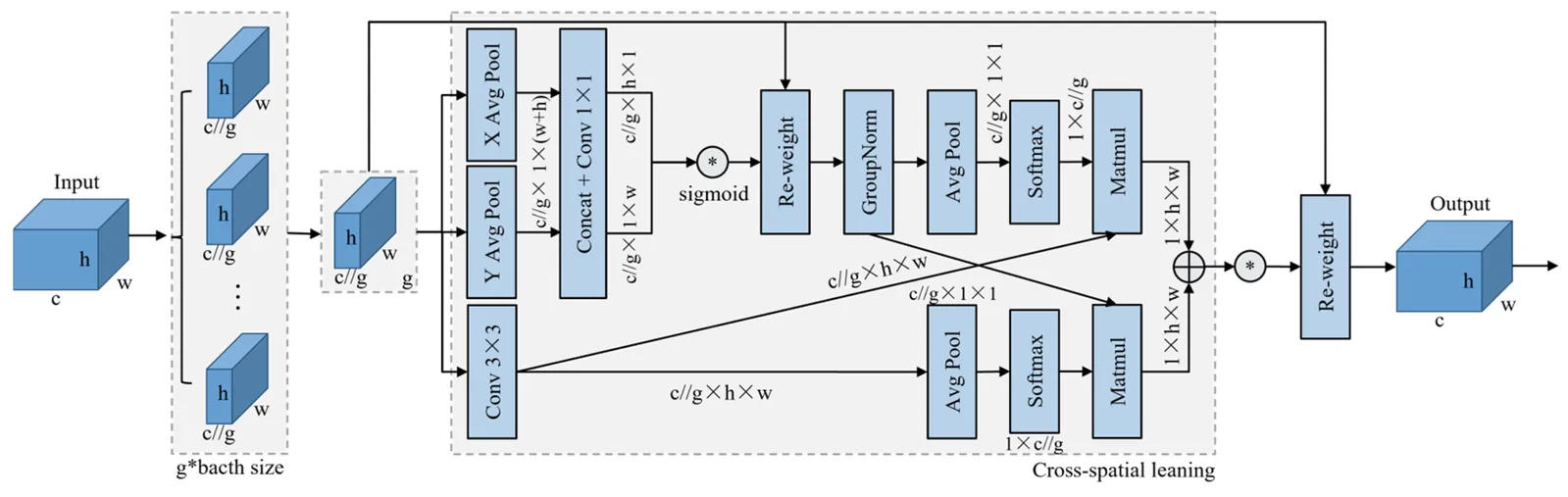
The detailed structure of the embedded EMA module.

The EMFE starts with channel dimension adjustment for input features of dimension $h\times w\times c_{in}$. The refined features are then fed into the embedded EMA to implement global adaptive feature reweighting. The internal structure of EMA is depicted in Figure 3. Distinct from conventional channel attention schemes that model channel dependencies in isolation, EMA leverages a cross-spatial learning paradigm. By grouping feature channels and capturing spatial cross-correlations among channel groups, EMA achieves adaptive enhancement of task-related feature responses without incurring heavy computational burdens. Such a mechanism suppresses background noise and invalid modal interference, creating favorable conditions for subsequent lightweight multi-scale feature modeling.

Following attention modulation, the feature tensor is evenly divided into two branches along the channel dimension via the Split operation, where each branch occupies $0.5c_{out}$ channels. One branch acts as a residual shortcut to retain primitive feature information, whereas the other branch undergoes multi-scale feature aggregation through cascaded faster blocks. As demonstrated in the enlarged subgraph of Figure 2, each faster block adopts PConv as its core operator. Instead of executing convolution over all channels, PConv computes features on partial channels and propagates the rest via identity mapping, thereby cutting computational costs compared with standard convolution. The outputs of PConv are further processed by stacked convolution layers combined with batch normalization and ReLU activation to introduce nonlinear transformation.

After multi-stage feature aggregation in faster blocks, the multi-scale branch and shortcut branch are concatenated along the channel dimension. A subsequent convolution operation unifies the channel number of fused features to $c_{out}$, finishing the forward inference pipeline of EMFE. Owing to the lightweight design of EMA and the PConv-equipped faster block, EMFE effectively curbs the computational and parameter overhead of single-modal feature extraction, accelerating the inference speed of the dual-branch backbone. The introduced EMA enables dynamic channel-wise weight allocation, guiding the model to concentrate on semantic features critical for pedestrian detection rather than trivial background patterns. By generating refined multi-scale representations, EMFE supports feature learning under unstable illumination and cluttered scenes.

### 2.3. Feature Alignment Restoration Module

**Figure 4:**
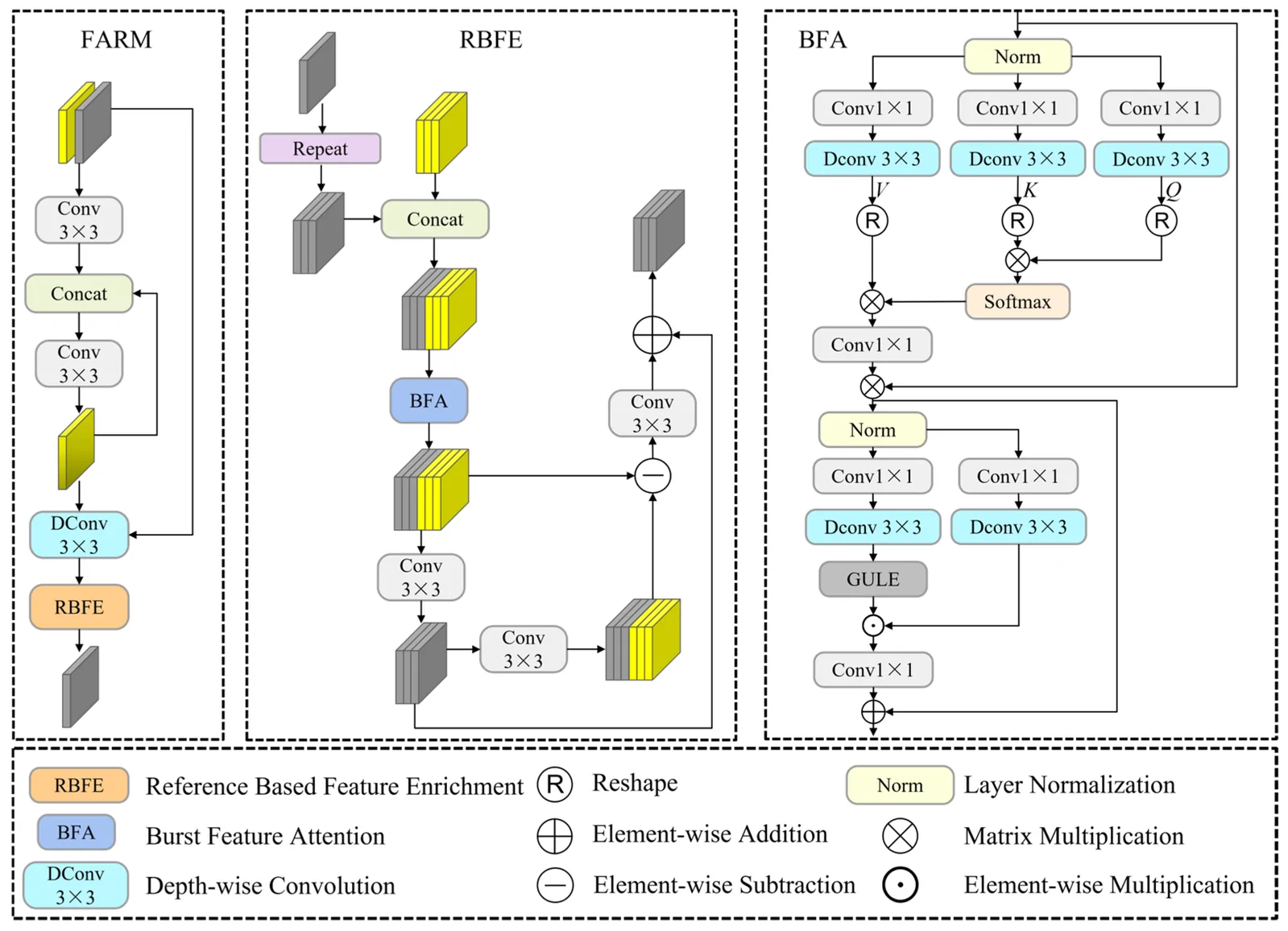
Structure of the proposed FARM.

RGB and thermal-infrared modalities present distinct discrepancies in feature distributions, which arise from the inherent spectral differences between RGB and thermal-infrared sensors. To tackle the challenges of cross-modal feature extraction and spatial alignment, we propose the FARM. Nevertheless, the alignment capability of this module is only applicable to cross-modal image pairs that are roughly or weakly registered. In addition, FARM achieves high-precision cross-modal feature calibration with lightweight computational overhead. Its detailed hierarchical architecture is illustrated in Figure 4.

The overall architecture of FARM consists of a pre-processing branch built on DConv and RBFE. Inside RBFE is embedded a BFA. A series of fundamental operators, including feature replication via repeat, channel concatenation, layer normalization, DConv and element-wise arithmetic operations, jointly complete cross-modal feature alignment, enhancement and restoration.

At the FARM input stage, paired RGB and infrared feature maps are separately fed into a standard convolutional layer for preliminary local feature extraction. The two groups of unimodal features are aggregated through concatenation to realize initial cross-modal information interaction. Afterwards, DConv is adopted to capture fine-grained local spatial cues with fewer parameters, enriching shallow feature representations without introducing excessive computational cost. The preprocessed fused feature is then delivered to the RBFE sub-module for reference-guided feature enhancement, and the transformation process can be formulated as Equation (1):

(1) $$F_{out}=\mathrm{RBFE}\left({\mathrm{DConv}}_{3\times 3}\left(\mathrm{Concat}\left({\mathrm{Conv}}_{3\times 3}\left(F_{in}\right),{\mathrm{Conv}}_{3\times 3}\left({\mathrm{Conv}}_{3\times 3}\left(F_{in}\right)\right)\right),F_{in}\right)\right),$$

Within the RBFE unit, visible and infrared feature maps are expanded through the Repeat operation, followed by channel concatenation to fully aggregate complementary information from two modalities. The fused multi-spectral feature is subsequently sent to the embedded BFA attention unit for deep feature optimization. The BFA unit constructs cross-modal correlation by building query, key and value branches. Combined with the built-in GELU unit, BFA implements pixel-level spatial offset calibration for cross-modal features and narrows the feature distribution gap caused by spectral inconsistency. Meanwhile, cross-spatial feature interaction enables feature maps to capture long-range contextual dependencies vital for pedestrian localization. After feature refinement via BFA, the optimized feature $F_{bfa}$ is obtained. Feature maps before and after attention processing perform element-wise subtraction and addition operations, as described in Equations (2) and (3):

(2) $$F=\mathrm{BFA}\left(\mathrm{Concat}\left(\mathrm{Repeat}\left(F_{vi}\right),\mathrm{Repeat}\left(F_{ir}\right)\right)\right),$$

(3) $$\hat{F}=\mathrm{Add}\left(\mathrm{Sub}\left({\mathrm{Conv}}_{3\times 3}\left({\mathrm{Conv}}_{3\times 3}\left(F\right)\right),F\right),{\mathrm{Conv}}_{3\times 3}\left(F\right)\right),$$

During feature alignment within BFA, layer normalization is deployed after each convolution block to regularize feature distributions. This stabilizes model training, prevents gradient vanishing or explosion, and accelerates convergence. Following the two-path feature interaction inside BFA, features pass through successive standard convolution layers for channel adjustment and feature refinement. Finally, the aligned, restored and enhanced cross-modal feature map is output as the result of the FARM.

Benefiting from the collaborative design of DConv, RBFE and BFA, FARM simultaneously realizes cross-modal spatial alignment, multi-spectral feature enrichment and global feature distribution optimization functions. The module effectively unifies feature consistency between RGB and infrared modalities, mitigates feature deviation induced by illumination variation, weather interference and background clutter. With DConv as the basic operator, the module balances representation capability and computational cost, making it suitable for real-time pedestrian detection tasks.

### 2.4. Dynamic Multi-Kernel Channel Attention

**Figure 5:**
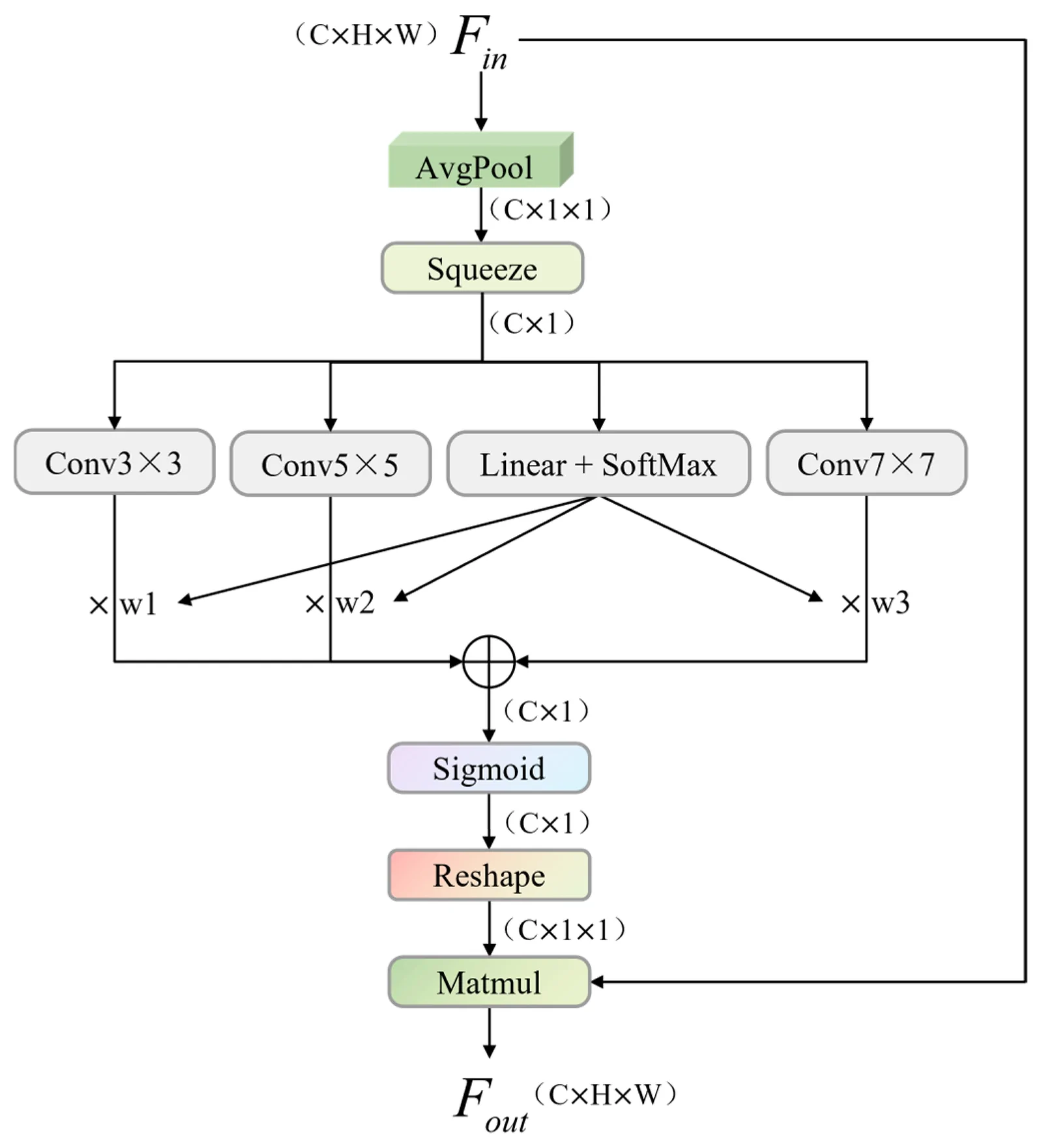
Structure of the proposed DMCA module.

To address the challenge of drastic scale variation among targets and the difficulty in adaptively balancing multi-scale features within RGB-infrared pedestrian detection scenarios, DMCA is proposed. The complete structural diagram of DMCA is illustrated in Figure 5. The core design philosophy of this module is to introduce parallel branches equipped with convolution kernels of diverse sizes to extract multi-scale spatial information and construct a dynamic weight allocation mechanism for adaptive fusion of feature channels at different scales.

Let the input feature map be $F_{in}\in {\mathbb{R}}^{C\times H\times W}$. The forward propagation pipeline of the DMCA module is elaborated as follows. First, global average pooling (AvgPool) is applied to the input features to aggregate global channel statistics, followed by a squeeze operation to eliminate redundant spatial dimensions and generate a compact one-dimensional channel descriptor F. The computation is defined in Equation (4):

(4) $$F=\mathrm{Squeeze}\left(\mathrm{AvgPool}\left(F_{in}\right)\right)\in {\mathbb{R}}^{C\times H\times W}$$

The squeezed channel descriptor F is fed into multiple parallel branches, including three multi-kernel convolution branches and an independent weight generation branch. The convolution branches adopt 3 × 3, 5 × 5 and 7 × 7 convolution kernels, respectively, to extract scale-specific features under different receptive fields. Each multi-scale feature branch is multiplied by its corresponding dynamic weight $w_{1}$, $w_{2}$ and $w_{3}$ to realize scale-aware feature weighting, as formulated in Equation (5):

(5) $$F_{k\times k}={\mathrm{Conv}}_{k\times k}\left(F\right)\times w_{k},k\;\in \left\{3,5,7\right\}$$

where k denotes the convolution kernel. The core innovation of DMCA lies in its dynamic weight generation mechanism. The compressed channel descriptor F passes through a linear transformation layer and a softmax activation function to automatically generate normalized importance weights $w_{1},w_{2},w_{3}$ for the three multi-scale branches. The Softmax operation guarantees that the sum of all weights equals 1, achieving input-aware adaptive weight assignment for multi-scale features, which is shown in Equation (6):

(6) $$w_{1},w_{2},w_{3}=\mathrm{Softmax}\left(\mathrm{Linear}\left(F\right)\right),$$

After obtaining the weighted multi-scale feature maps $F_{3\times 3}$, $F_{5\times 5}$ and $F_{7\times 7}$, these feature maps are summed via the add operation. The fused feature is fed into a sigmoid activation function to generate normalized channel attention weights. Next, a reshape operation is adopted to adjust the dimension of attention weights to match the size of the original input feature map $F_{in}$. Finally, matrix multiplication is performed between the reshaped attention weights and the input feature $F_{in}$ to complete channel-wise feature enhancement, and the final output feature $F_{out}$ is obtained. The complete calculation process is defined by Equation (7):

(7) $$F_{out}=F_{in}⊙\mathrm{Reshape}\left(\mathrm{Sigmoid}\left(\mathrm{Add}\left(F_{3\times 3},F_{5\times 5},F_{7\times 7}\right)\right)\right),$$

Benefiting from the dynamic multi-kernel adaptive weight allocation mechanism, DMCA can flexibly adjust the contribution ratio of feature channels at different scales according to the content of input images, which enhances the model’s adaptability to multi-scale targets. This multi-scale channel fusion strategy can fully mine differentiated spatial context information corresponding to small, medium and large objects, and exhibits prominent advantages in complex multi-spectral scenes with cluttered backgrounds and uneven target scale distribution. Meanwhile, the module reinforces the attention screening mechanism from the channel dimension, automatically suppressing the responses of irrelevant and redundant feature channels while continuously amplifying discriminative features beneficial to pedestrian detection. DMCA is general and portable; it can be embedded into mainstream detection architectures and extended to other multi-scale visual tasks beyond multispectral pedestrian detection.

## 3. Results and Discussion

### 3.1. Experimental Settings

#### 3.1.1. Experimental Settings and Datasets

EFDNet is developed on the PyTorch 2.0.0 framework. For fair evaluation, an IoU threshold of 0.5 is adopted to determine positive samples. Samples with IoU exceeding this threshold are treated as positive, and the rest as negative. This assignment rule is consistent across all compared methods. All experiments are performed on four TITAN X GPUs, with the software stack comprising Ubuntu 22.04, Python 3.9.1 and CUDA Toolkit 11.8. The network is optimized via SGD. We configure an initial learning rate of 0.02, a batch size of 16, a momentum of 0.937, and a weight decay of 0.0005, and train the model for 120 epochs in total.

To validate the effectiveness of EFDNet, comparative evaluations are performed over three public infrared–visible pedestrian datasets, including KAIST [22], LLVIP [23], and FLIR [24]. For the KAIST dataset, the training set adopts the improved annotation version proposed in AR-CNN, containing 8963 image pairs, while the test set uses the revised annotation version with 2252 images. The LLVIP dataset provides 12,025 image pairs for training and 3463 pairs for testing. The FLIR dataset employs an aligned and cleaned version, containing 4129 training image pairs and 1013 testing image pairs. All models are trained and tested on the full multi-class sets of FLIR and LLVIP, while only the detection metrics of the pedestrian class are reported for comparison.

#### 3.1.2. Evaluation Metrics

Based on the aforementioned datasets, different evaluation metrics are applied. On the KAIST dataset, log-average miss rate (L-AMR) is adopted as the evaluation metric. This metric is calculated by averaging the miss rates sampled at false positives per image (FPPI) points ranging from 10^−2^ to 10^2^ (denoted as MR^−2^). For the FLIR and LLVIP datasets, standard evaluation metrics from the MS-COCO benchmark, which are widely recognized in the object detection community, are utilized to measure model performance. Higher values of these metrics indicate superior detection performance. Specifically, mAP_50–95_ refers to the mean average precision computed at intersection-over-union (IoU) thresholds spanning from 0.5 to 0.95 with an interval of 0.05; mAP_50_ denotes the average precision at an IoU threshold of 0.5, while mAP_75_ represents the average precision when the IoU threshold is set to 0.75.

### 3.2. Results and Analysis

#### 3.2.1. Results on KAIST Dataset

Based on the experimental settings described above, a comprehensive comparison was conducted on the KAIST dataset between the proposed EFDNet and a series of state-of-the-art multi-modal pedestrian detection methods, including Fusion RPN + BF [25], IAF-RCNN [26], IATDNN + IASS [27], CIAN [28], MSDS-RCNN [29], AR-CNN [30], AMFD [10], MBNet [31], MLPD [32], OctNet [33] and HAFNet [34]. Table 1 summarizes the quantitative results in terms of L-AMR across different scene splits as well as inference latency. The reported inference speeds are collected from original publications and measured on different GPUs and software frameworks. Direct quantitative comparison of latency across methods is not strictly fair, and these speed values are provided only for reference.

As illustrated in Table 1, EFDNet achieves superior overall performance with the lowest miss rate of 6.73% on the reasonable-all subset. In particular, this method obtains a prominent miss rate of 4.11% for nighttime scenarios, which outperforms all comparative models. This remarkable nighttime detection capability demonstrates that the designed FARM effectively reduces cross-modal feature discrepancies and fully exploits infrared thermal complementary information under low-light environments. For daytime scenes, EFDNet achieves a miss rate of 8.03%. HAFNet and MLPD achieve better daytime performance with miss rates of 7.68% and 7.96%, respectively. The minor performance gap is mainly attributed to the lightweight architecture of EFDNet, which pursues balanced accuracy and efficiency rather than excessive parameter stacking. In terms of inference speed, EFDNet processes each image within 0.04 s on a TITAN X GPU, achieving competitive real-time capability among most compared methods. Compared with the much faster MLPD, which achieves 0.012 s but yields a higher nighttime miss rate of 6.95%, EFDNet achieves a better trade-off between detection accuracy and computational efficiency.

**Figure 6:**
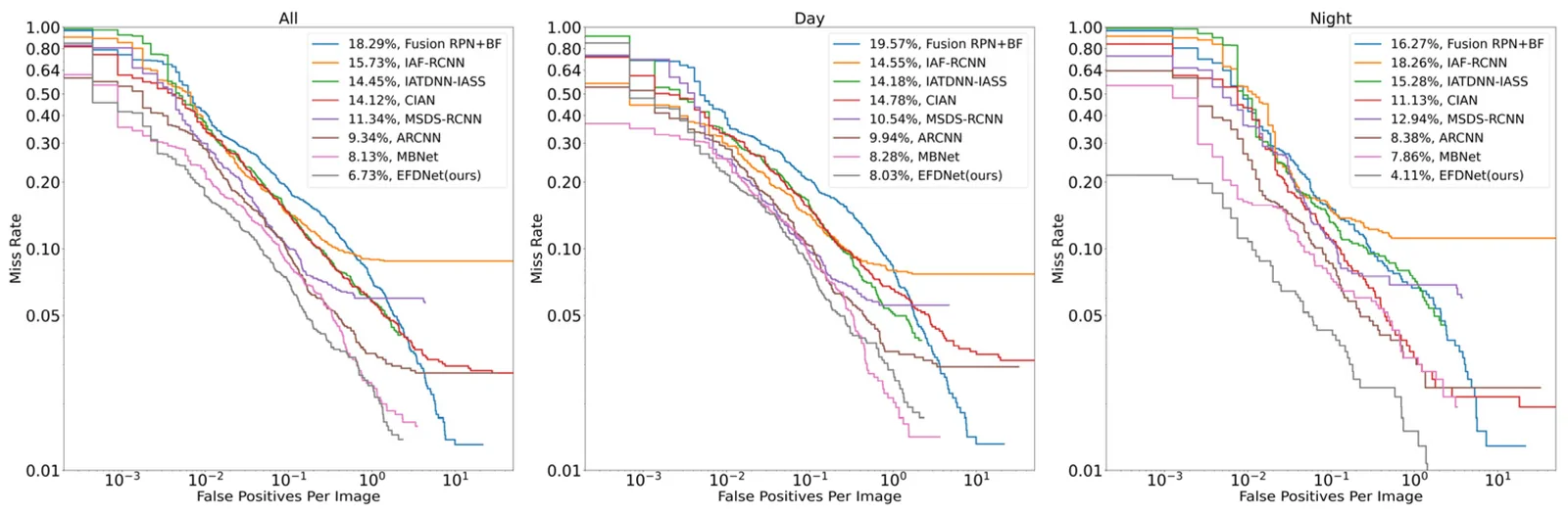
Model comparison results under the reasonable evaluation protocol.

Figure 6 plots the miss rate-FPPI curves of EFDNet and other multimodal pedestrian detectors on the KAIST reasonable subset under an IoU threshold of 0.5, covering all-day, daytime and nighttime scenarios. Consistent with the observation from Table 1, EFDNet achieves leading performance for all-day and nighttime samples. Compared with MLPD, EFDNet obtains a notable reduction in miss rate at night and achieves better results on the all-day subset. The obvious performance gain proves that, equipped with FARM, EFDNet can extract highly discriminative pedestrian features in dim environments by sufficiently utilizing complementary infrared information.

**Figure 7:**
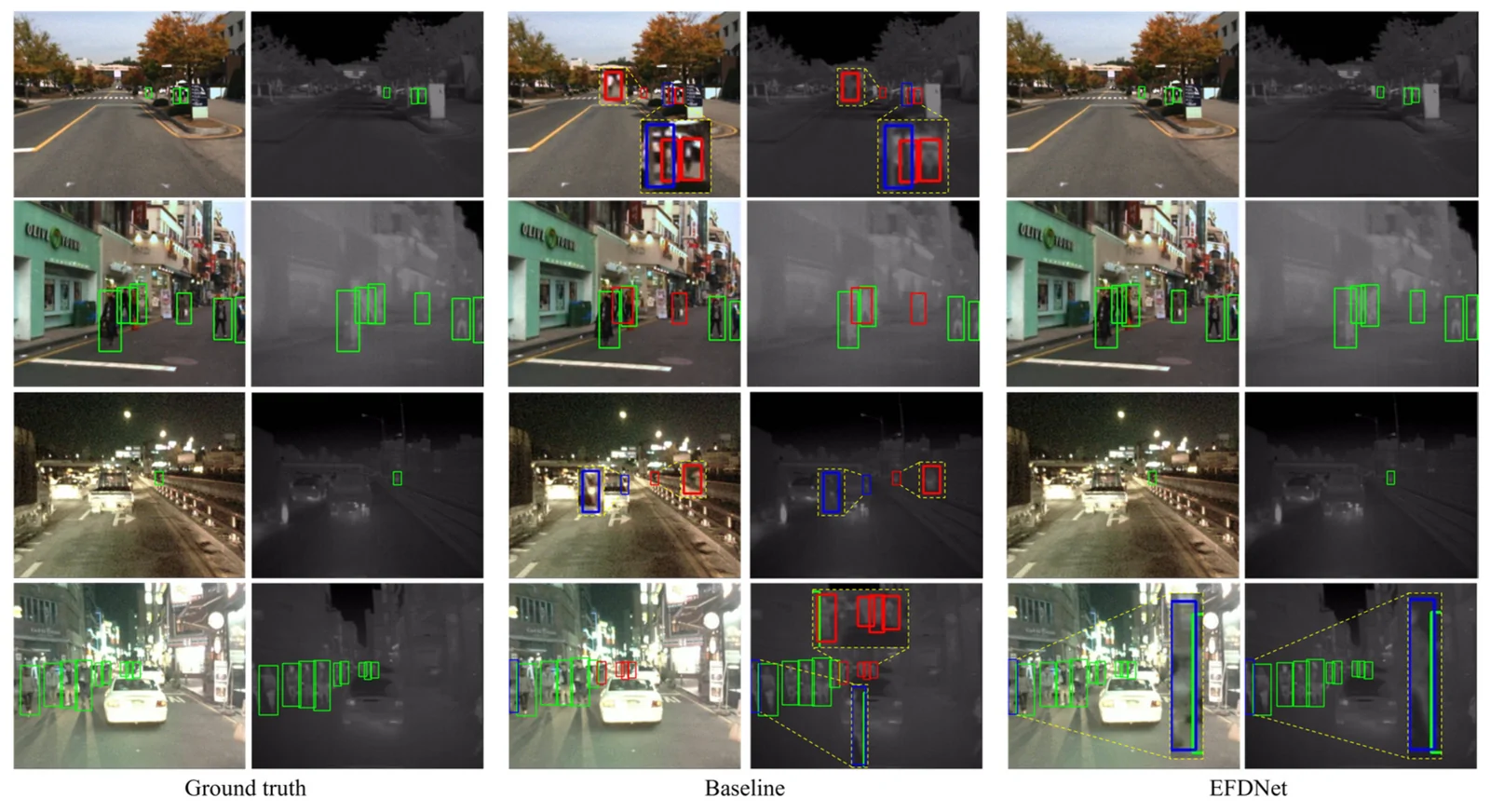
Visual comparison of pedestrian detection results on the KAIST dataset. Green boxes: true positives; blue boxes: false positives; red boxes: missed pedestrians. From (**left**) to (**right**): ground truth, baseline model, and the proposed EFDNet.

Figure 7 provides visual comparisons among ground truth, the baseline model and EFDNet on KAIST samples. In the visualization results, green boxes represent true positives, blue boxes correspond to false positives, and red boxes denote missed pedestrian targets. Visual evidence reveals that EFDNet outperforms the baseline across complicated road scenes, especially for distant small pedestrians and nighttime low-light cases. In daytime street scenes, the baseline frequently misses distant and peripherally distributed pedestrians, which are marked by numerous red boxes. By contrast, EFDNet successfully detects these difficult samples, showing strong robustness against target scale variation and irregular spatial distribution. The performance advantage is further amplified at night. The limited visibility of RGB images causes heavy missed detections for the baseline. Benefiting from sufficient utilization of infrared thermal information, EFDNet effectively reduces missed targets and restrains false positives simultaneously. In addition, the predicted bounding boxes of EFDNet overlap better with the ground truth, reflecting higher localization accuracy.

#### 3.2.2. Results on FLIR Dataset

Quantitative comparisons between EFDNet and a set of cross-modal detection approaches are conducted on the FLIR dataset, as listed in Table 2. The compared methods cover a variety of advanced models, including GAFF [35], CAF [36], CFT [37], CSAA [38], ICAFusion [39], Crossformer [40], two-stream YOLOv8 [41], MMI-Det [42], YOLO-MEDet [43] and Fusion-DETR [44]. EFDNet achieves the highest scores on both mAP_50_ and mAP_75_, reaching 82.8% and 36.6%, respectively, outperforming the second-best method by 1.3% and 0.8%. On the comprehensive metric mAP_50–95_, EFDNet attains 42.0%, which is lower than Fusion-DETR’s 44.3% but still surpasses other models such as CFT with 40.2% and MMI-Det with 40.5%. Overall, EFDNet demonstrates strong competitiveness in detection accuracy, particularly excelling under stricter localization requirements. Equipped with the EMFE module that aggregates global and local feature extraction branches, the model can capture global context and fine-grained local details synchronously. Such design enhances feature representation capability and boosts the recognition accuracy of pedestrian targets.

**Figure 8:**
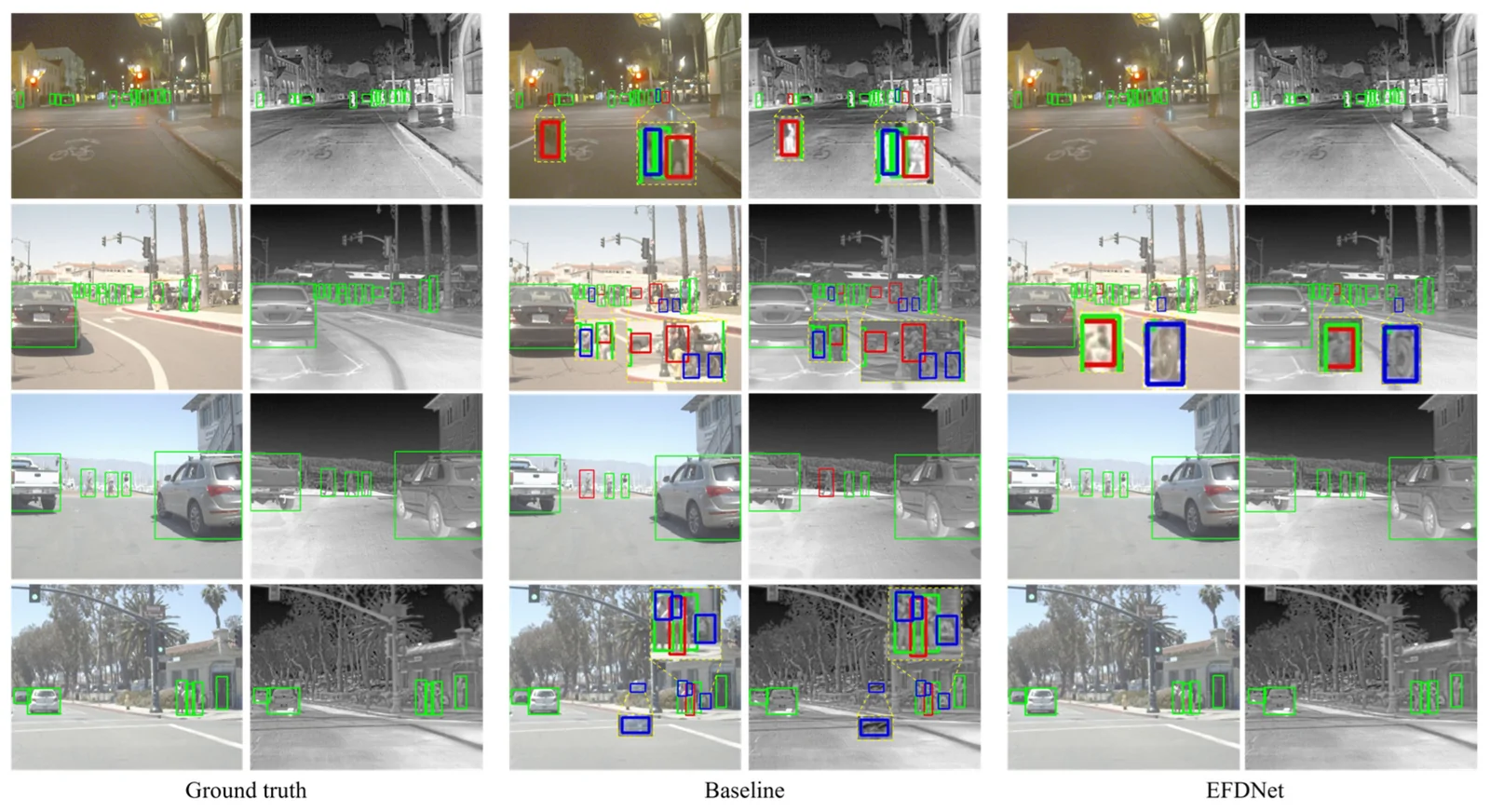
Visualization of pedestrian detection results on the FLIR dataset. Green boxes: true positives; blue boxes: false positives; red boxes: missed pedestrians. From (**left**) to (**right**): ground truth, baseline model, and the proposed EFDNet.

Figure 8 provides qualitative visual comparisons of ground truth, baseline predictions and EFDNet outputs on FLIR test samples, which further validates the advantages of the proposed method under various urban traffic scenarios.

In nighttime street scenes, low visibility in RGB images causes serious missed detections for the baseline. By sufficiently utilizing infrared thermal radiation information, EFDNet overcomes illumination constraints of visible images and achieves higher recall rates. For daytime scenes containing pedestrians and vehicles, the baseline suffers from both missed targets and false positives. Relying on effective cross-modal feature fusion, EFDNet can accurately separate pedestrians from vehicle backgrounds, maintaining high recall while suppressing false alarms. Partially occluded and distant pedestrians in parking lots and open roads bring great challenges to the baseline and trigger massive missed detections. In contrast, EFDNet exploits complementary cross-modal cues to robustly tackle occlusion and scale variation. The bounding boxes predicted by EFDNet exhibit higher consistency with ground truth annotations.

#### 3.2.3. Results on LLVIP Dataset

Table 3 summarizes quantitative comparisons between EFDNet and mainstream detection algorithms on the LLVIP dataset, including GAFF [35], CASS [38], two-stream YOLOv8 [41], TFDet [45], CFT [37], CCIFNet [46], MCOR [47] and Fusion-DETR [44]. EFDNet achieves an mAP_50_ of 97.3%, an mAP_75_ of 79.0% and an overall mAP_50–95_ of 68.2%. The LLVIP dataset consists of relatively simple scenes, and most competing methods obtain an mAP_50_ exceeding 90%, while state-of-the-art approaches surpass 97%. Such results lead to small performance gaps under the loose IoU threshold of 0.5. Accordingly, stricter metrics including mAP_75_ and overall mAP_50–95_ are more suitable for evaluating localization precision. EFDNet achieves the best performance on both mAP_75_ and overall mAP_50–95_. It outperforms the second-ranked MCOR by 5.3% on mAP_75_ and the second-ranked Fusion-DETR by 1.3% on overall mAP_50–95_. More notably, compared with CFT, EFDNet achieves improvements of 6.1% on mAP_75_ and 4.6% on mAP_50–95_. These results prove that the proposed model possesses stronger spatial perception and accurate boundary regression capability for pedestrian targets.

**Figure 9:**
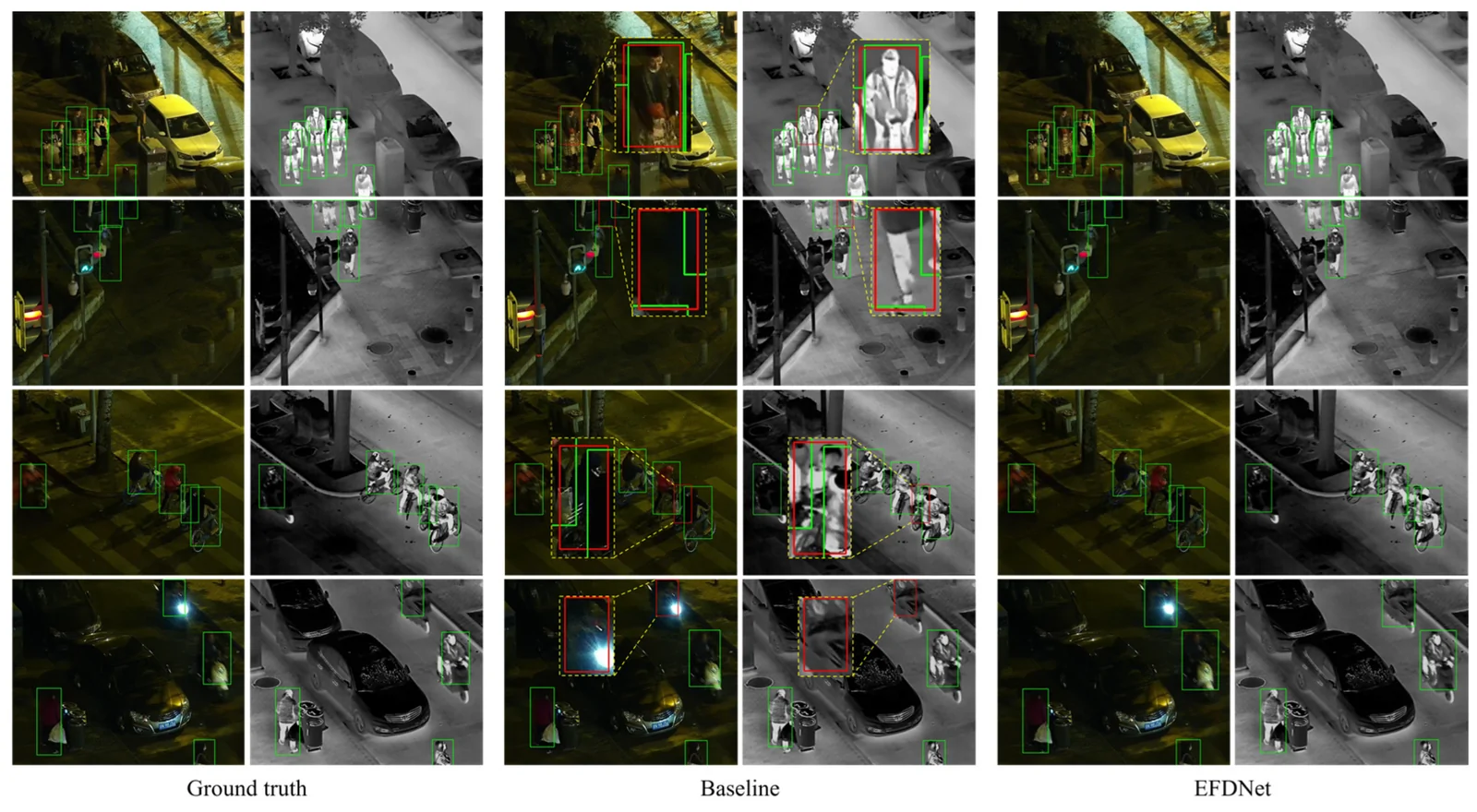
Visualization of pedestrian detection results on the LLVIP dataset. Green boxes: true positives; red boxes: missed pedestrians. From (**left**) to (**right**): ground truth, baseline model, and the proposed EFDNet.

Figure 9 provides qualitative visual comparisons of detection outputs among ground truth, the baseline model and EFDNet on the LLVIP test set. The baseline shows obvious defects under challenging conditions such as low illumination, target occlusion and small objects, manifested by numerous missed targets and blue false-positive boxes. This observation reveals inherent limitations of conventional detectors in feature extraction and context modeling. By contrast, equipped with feature decoupling and deep cross-modal fusion modules, EFDNet improves the perception of multi-scale targets and generates denser and more accurate green true-positive bounding boxes, demonstrating its powerful ability to detect pedestrian instances.

In low-light nighttime scenes, the baseline suffers from frequent false positives and missed detections. It fails to distinguish foreground pedestrians from cluttered backgrounds and generates low-discriminative features under noisy conditions. Benefiting from feature enhancement and decoupling operations, EFDNet effectively separates target features from background interference and suppresses false alarms. Meanwhile, multi-scale feature fusion strengthens the model’s capacity to capture subtle edge and texture information under dim lighting and effectively reduces missed detections. For crowded scenes with overlapping pedestrians and vehicles, limited receptive fields and insufficient context information prevent the baseline from precisely localizing occluded targets, resulting in incomplete and offset bounding boxes. With the elaborate design of the EMFE, FARM and DMCA modules, EFDNet accurately locates each pedestrian and achieves favorable boundary regression. Its predicted bounding boxes are closely aligned with ground truth annotations. These visualization results confirm the strong scene understanding capability of the proposed network.

### 3.3. Ablation Study

To verify the effectiveness of the proposed EMFE, FARM and DMCA modules, ablation experiments are carried out on the LLVIP, FLIR and KAIST datasets for quantitative analysis.

#### 3.3.1. Quantitative Results

As illustrated in Table 4, Table 5 and Table 6, the complete EFDNet achieves the optimal overall performance across the three datasets and nearly all evaluation metrics. Compared with the baseline model, EFDNet consistently reduces the L-AMR on the KAIST dataset and improves precision, recall, mAP_50_, mAP_75_, and mAP_50–95_ on FLIR and LLVIP. The cumulative performance gains across different datasets demonstrate that the three designed modules can cooperate effectively under diverse scene conditions. The superior performance of EFDNet verifies the overall effectiveness and generalization capability of the proposed multi-module collaborative framework.

The EMFE module delivers steady and effective feature enhancement performance. It improves detection accuracy in complex daytime and urban scenes by strengthening multi-scale feature representation and enhancing the model’s perception of occluded and deformed pedestrian hard samples. On the KAIST daytime subset, EMFE independently reduces L-AMR by 3.96, achieving the largest single-module daytime gain. Nevertheless, the improvement of EMFE is relatively limited in nighttime scenarios, bringing only a 1.42 drop in nighttime L-AMR on KAIST. This phenomenon arises from the lack of reliable visible information, which restricts the quality of multi-scale feature learning and indicates that a standalone EMFE cannot fully compensate for the deficiency of infrared feature discrimination.

The FARM effectively alleviates cross-modal feature discrepancies caused by spectral mismatch and modal differences. Through dual-branch feature interaction, FARM realizes feature alignment and compensation between visible and infrared modalities, thereby reducing missed and false detections. On the FLIR dataset, FARM improves mAP_50_ by 3.2%, which outperforms the other two individual modules. Stable and balanced performance gains can be observed across all datasets, demonstrating that cross-modal alignment is essential for maintaining consistent detection accuracy under diverse illumination and scene conditions.

The DMCA module exhibits prominent scene-adaptive optimization capability. As a multi-scale extension of ECA, it adopts three parallel convolutions to model multi-scale channel dependencies and dynamically adjusts channel weights. DMCA enhances the model’s sensitivity to blurred, small, and low-contrast infrared targets. On the KAIST nighttime subset, DMCA reduces L-AMR by 3.00 and achieves the best single-module performance for night detection, which validates that dynamic multi-kernel channel attention can effectively cope with drastic pedestrian scale variations and low-light environment challenges.

Table 7 summarizes the parameter count and GFLOPs of different module combinations. EMFE and DMCA introduce negligible overhead: EMFE only increases the model parameters from 2.37 M to 2.38 M and GFLOPs from 6.7 to 6.8, with an inference latency of 35.9 ms. DMCA also brings nearly no extra computational burden. In contrast, FARM accounts for the major increase in model complexity, raising parameters to around 5.33 M and GFLOPs to 12.8; its inference latency is 36.2 ms. Although FARM imposes higher computational cost, it provides critical cross-modal alignment gains. EMFE and DMCA remain lightweight, which makes the full EFDNet achieve favorable detection accuracy without excessive computational overhead.

Furthermore, the performance improvements brought by EMFE, FARM, and DMCA are not linearly additive. Individual modules partially overlap in the failure cases they correct, resulting in moderate overall gains when all modules are integrated. Nevertheless, the three modules target distinct model defects: EMFE strengthens low-level multi-scale feature representation, FARM resolves cross-modal spatial misalignment, and DMCA optimizes multi-scale channel feature recalibration. Their combination compensates for the limitations of individual components and yields comprehensive performance improvements in complex multi-spectral scenarios.

#### 3.3.2. Visualization Results

**Figure 10:**
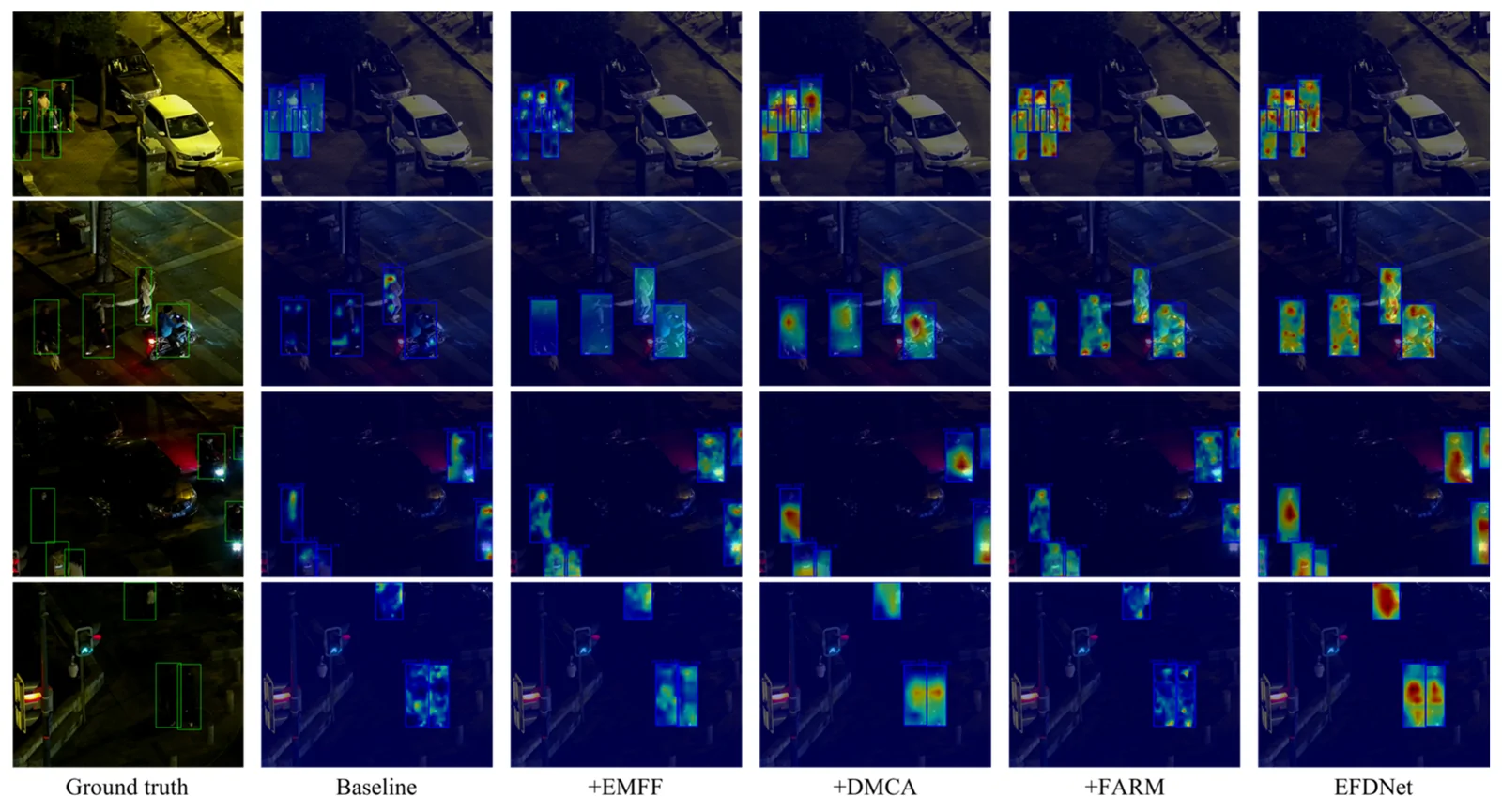
Heatmap comparison of ablation experiments on the LLVIP dataset.

To intuitively analyze the attention preference of different network variants, class activation heatmaps derived from nighttime samples of the LLVIP dataset are illustrated in Figure 10. The columns from left to right correspond to ground truth, the baseline model, the baseline integrated with EMFE, the baseline integrated with DMCA, the baseline integrated with FARM, and the complete EFDNet.

For the baseline model, the network exhibits dispersed attention preference. It cannot consistently focus on pedestrian bodies, and considerable attention is diverted to irrelevant background regions. After embedding the EMFE module, the network starts to prioritize areas containing pedestrians, but it still fails to capture the full range of pedestrian targets. When only DMCA is introduced, the network strengthens its focus on target regions, yet partial attention drifts away from actual pedestrian locations. The addition of FARM further improves the localization of attention: the network becomes more sensitive to pedestrian boundaries and aligns cross-modal features more precisely, leading to tighter and more coherent activation around pedestrian instances.

Benefiting from the joint effect of EMFE, FARM and DMCA, EFDNet achieves the most desirable attention behavior. The network concentrates its attention tightly on pedestrian instances, while allocating minimal attention to cluttered backgrounds. This observation demonstrates that EMFE builds a solid foundation for multi-scale feature extraction, FARM corrects feature misalignment between two modalities and DMCA amplifies feature responses associated with targets. The coordination of these three modules guides the network to pay sustained and accurate attention to pedestrians rather than background noise.

#### 3.3.3. Robustness to Cross-Modal Misalignment

**Figure 11:**
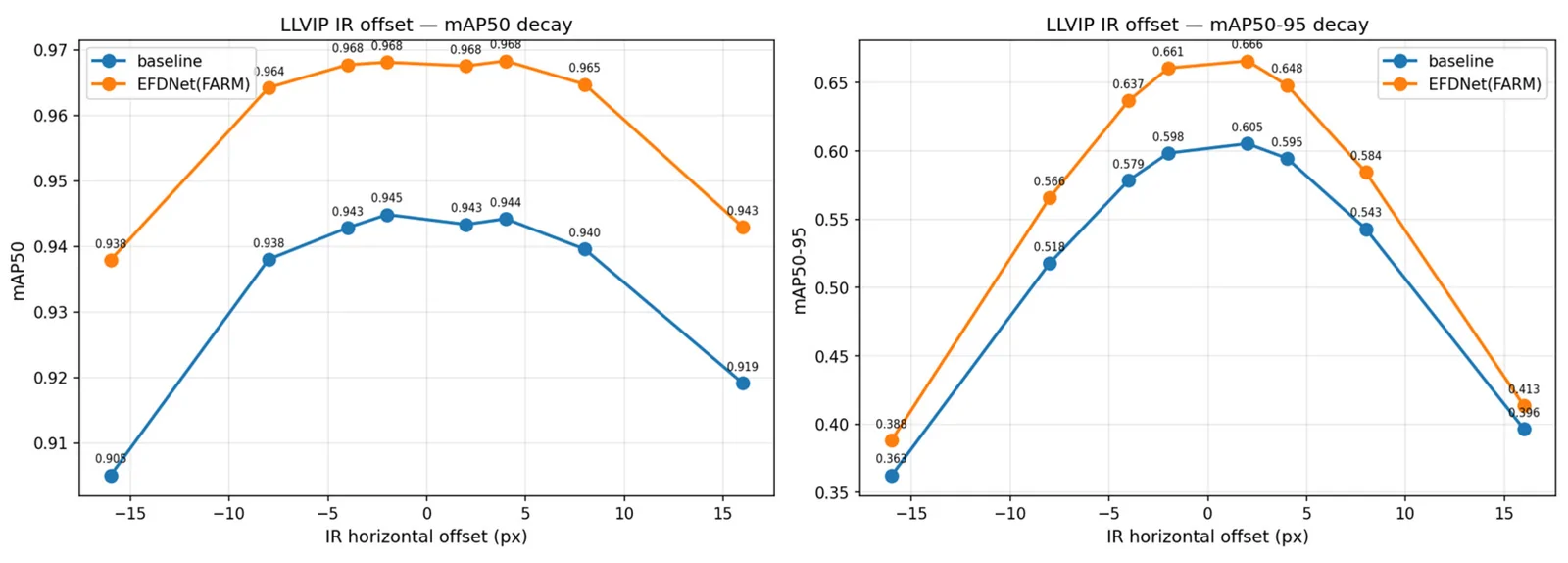
mAP decay curves under horizontal infrared offset on LLVIP.

To verify the alignment capability of FARM under cross-modal misalignment, we construct synthetic misalignment by horizontally shifting infrared images in the LLVIP validation set by ±2, ±4, ±8, ±16 pixels. Table 8 lists the mAP_50_ and mAP_50–95_ of the baseline and the model equipped with FARM under different offsets. Figure 11 presents the corresponding performance decay curves.

It can be observed that detection performance gradually degrades as the offset increases. The model with FARM consistently outperforms the baseline at all offset magnitudes. The performance gap becomes larger under severe misalignment, demonstrating that FARM effectively mitigates cross-modal spatial misalignment and improves robustness to registration error.

## 4. Conclusions

This paper proposes an end-to-end RGB-infrared multispectral pedestrian detection network, termed EFDNet, to tackle inherent challenges including redundant feature representation, cross-modal feature misalignment, and scale-variation sensitivity. The overall architecture incorporates three dedicated modules: the EMFE module for multi-scale feature modeling, the FARM for cross-modal feature calibration, and the DMCA module for adaptive feature enhancement.

The EMFE module is designed to strengthen the discriminative representation of edge and texture cues while constraining computational overhead, thereby generating refined multispectral features to support subsequent cross-modal fusion. Built on a residual alignment-fusion paradigm, the FARM differs from conventional two-stage pipelines that perform fusion prior to feature calibration. Through internal dual-branch interactive learning, it achieves synchronous cross-modal alignment at both pixel and feature levels, effectively mitigating edge blurring and feature distribution deviation induced by infrared thermal halo interference. The DMCA module is further embedded to handle drastic scale variations in pedestrian targets. It leverages multi-scale parallel convolutions to capture spatial features across diverse scales and adopts lightweight auxiliary branches to dynamically generate data-adaptive attention weights. This design enables joint modulation across spatial scales and feature channels, improving detection stability for pedestrian targets of varying sizes and spatial distances.

Extensive experiments are carried out on three public benchmarks: KAIST, FLIR, and LLVIP. Quantitative results demonstrate that EFDNet achieves competitive performance on mainstream evaluation metrics, yielding superior detection accuracy and robustness under nighttime conditions and cluttered, complex scenarios. Ablation studies and heatmap visualizations further validate that EMFE, FARM, and DMCA form a hierarchically optimized detection framework through tight functional collaboration and complementary information interaction. Although this work has achieved encouraging performance, EFDNet still has several limitations that require further research. The current fixed network architecture lacks an adaptive adjustment mechanism for the complexity of different input scenarios, which may result in suboptimal computational efficiency in simple scenarios. Additionally, the existing fusion strategy is mainly optimized for clear-weather conditions, and its generalization capability under extreme weather conditions still needs in-depth exploration. Furthermore, this work assumes that the dual-modal image pairs are roughly or weakly aligned. Accordingly, future research will focus on three main directions: first, further reduce the computational cost of EFDNet to facilitate its efficient deployment on embedded mobile platforms; second, design robust multispectral fusion strategies for adverse weather conditions to promote the practical application of all-weather autonomous driving perception systems; third, for cases of extreme disparity or severe non-rigid distortion, explicit registration or optical flow guidance may be needed to better achieve dual-modal pedestrian detection.

## Figures and Tables

**Table 1:** Quantitative comparison on the KAIST dataset.

Method	Backbone	MR^−2^ (IoU = 0.5)			Platform	Speed (s)
		All	Day	Night		
Fusion RPN + BF [25]	VGG-16	18.29	19.57	16.27	MATLAB	0.800
IAF-RCNN [26]	VGG-16	15.73	14.55	18.26	TITAN X	0.210
IATDNN + IASS [27]	VGG-16	14.95	14.67	15.72	TITAN X	0.250
CIAN [28]	VGG-16	14.12	14.78	11.13	1080Ti	0.070
MSDS-RCNN [29]	VGG-16	11.34	10.54	12.94	TITAN X	0.220
AR-CNN [30]	VGG-16	9.39	9.94	8.38	1080Ti	0.120
AMFD [10]	ResNet-18	8.82	10.98	4.86	1080Ti	0.040
MBNet [31]	ResNet-50	8.13	8.28	7.86	1080Ti	0.070
MLPD [32]	VGG-16	7.58	7.96	6.95	2080Ti	**0.012**
OctNet [33]	VGG-16	7.15	**7.57**	6.27	RTX 3090	0.048
HAFNet [34]	CSPDarkNet	6.93	7.68	5.66	1080Ti	0.017
EFDNet (ours)	CSPDarkNet	**6.73**	8.03	**4.11**	TITAN X	0.040

Bold represents the best results.

**Table 2:** Quantitative comparison between EFDNet and other methods on the FLIR dataset.

Method	Modality	Backbone	mAP_50_	mAP_75_	mAP_50–95_
GAFF [35]	RGB + IR	ResNet18	72.9	32.9	37.5
CAF [36]	RGB + IR	CSPDarkNet	74.7	34.0	38.4
CFT [37]	RGB + IR	CFB	78.7	35.5	40.2
CSAA [38]	RGB + IR	ResNet50	79.2	-	41.3
ICAFusion [39]	RGB + IR	VGG16	79.2	-	41.4
Crossformer [40]	RGB + IR	ResNet50	79.3	-	42.1
Two-stream YOLOv8 [41]	RGB + IR	CSPDarknet	79.7	31.3	38.9
MMI-Det [42]	RGB + IR	MMI-Det	79.8	35.8	40.5
YOLO-MEDet [43]	RGB + IR	CSPDarknet	80.1	35.1	38.5
Fusion-DETR [44]	RGB + IR	ResNet101	81.5	-	**44.3**
EFDNet (ours)	RGB + IR	CSPDarknet	**82.** **8**	**36.** **6**	42.0

Bold represents the best results.

**Table 3:** mAP comparison between EFDNet and other approaches on the LLVIP dataset.

Method	Modality	Backbone	mAP_50_	mAP_75_	mAP_50–95_
GAFF [35]	RGB + IR	ResNet18	94.0	-	55.8
CASS [38]	RGB + IR	ResNet50	94.3	-	59.2
Two-stream YOLOv8 [41]	RGB + IR	CSPDarknet	95.4	73.2	63.7
TFDet [45]	RGB + IR	VGG16	96.0	**-**	59.4
CFT [37]	RGB + IR	CFB	97.5	72.9	63.6
CCIFNet [46]	RGB + IR	ResNet50	**97.6**	72.6	63.6
Fusion-DETR [44]	RGB + IR	ResNet101	97.2	-	66.9
MCOR [47]	RGB + IR	CSPDarknet	**97.6**	73.7	64.9
EFDNet (ours)	RGB + IR	CSPDarknet	97.3	**79.0**	**68.** **2**

Bold represents the best results.

**Table 4:** Ablation study of MR^−2^ on the KAIST dataset.

Baseline	EMFE	FARM	DMCA	MR^−2^ (IoU = 0.5)		
				All	Day	Night
✓				11.70	13.14	9.18
✓	✓			8.60 (−3.10)	9.18 (−3.96)	7.76 (−1.42)
✓		✓		8.98 (−2.72)	9.64 (−3.50)	7.68 (−1.50)
✓			✓	9.04 (−2.66)	10.20 (−2.94)	6.18 (−3.00)
✓	✓	✓		8.52 (−3.18)	8.88 (−4.26)	7.72 (−1.46)
✓	✓		✓	8.04 (−3.66)	9.42 (−3.72)	5.04 (−4.14)
✓		✓	✓	8.13 (−3.57)	8.77 (−4.37)	7.13 (−2.05)
✓	✓	✓	✓	**6.73 (−4.97)**	**8.03 (−5.11)**	**4.11 (−5.07)**

Bold represents the best results.

**Table 5:** Ablation experimental results on the FLIR dataset.

Baseline	EMFE	FARM	DMCA	Precision	Recall	mAP_50_	mAP_75_	mAP_50–95_
✓				82.4	67.7	79.7	31.3	38.9
✓	✓			83.4 (+1.0)	69.4 (+1.7)	81.6 (+1.9)	35.6 (+4.3)	40.8 (+1.9)
✓		✓		83.6 (+1.2)	72.7 (+5.0)	**82.9 (+3.2)**	35.7 (+4.4)	41.5 (+2.6)
✓			✓	81.7 (−0.7)	**73.9 (+6.2)**	82.2 (+2.5)	34.1 (+2.8)	41.0 (+2.1)
✓	✓	✓		84.0 (+1.6)	70.6 (+2.9)	82.6 (+2.9)	36.2 (+4.9)	41.7 (+2.8)
✓	✓		✓	83.9 (+1.5)	71.0 (+3.3)	81.7 (+2.0)	35.0 (+3.7)	41.0 (+2.1)
✓		✓	✓	83.1 (+0.7)	72.3 (+4.6)	82.7 (+3.0)	**37.0 (+5.7)**	41.9 (+3.0)
✓	✓	✓	✓	**84.1 (+1.7)**	72.5 (+4.8)	82.8 (+3.1)	36.6 (+5.3)	**42.0 (+3.1)**

Bold represents the best results.

**Table 6:** Ablation experimental results on the LLVIP dataset.

Baseline	EMFE	FARM	DMCA	Precision	Recall	mAP_50_	mAP_75_	mAP_50–95_
✓				93.9	89.3	95.4	73.2	63.7
✓	✓			94.4 (+0.5)	92.2 (+2.9)	96.7 (+1.3)	77.6 (+4.4)	67.2 (+3.5)
✓		✓		94.5 (+0.6)	92.5 (+3.2)	96.9 (+1.5)	78.1 (+4.9)	67.2 (+3.5)
✓			✓	95.2 (+1.3)	91.2 (+1.9)	96.6 (+1.2)	77.5 (+4.3)	66.7 (+3.0)
✓	✓	✓		94.6 (+0.7)	93.0 (+3.7)	97.0 (+1.6)	78.7 (+5.5)	68.2 (+4.5)
✓	✓		✓	94.6 (+0.7)	92.0 (+2.7)	96.8 (+1.4)	78.4 (+5.2)	67.3 (+3.6)
✓		✓	✓	94.5 (+0.6)	93.0 (+3.7)	97.1 (+1.7)	78.6 (+5.4)	**68.3 (+4.6)**
✓	✓	✓	✓	**95.4 (+1.5)**	**93.2 (+3.9)**	**97.3 (+1.9)**	**79.0 (+5.8)**	68.2 (+4.5)

Bold represents the best results.

**Table 7:** Computational complexity comparison of different modules on the dataset.

Baseline	EMFE	FARM	DMCA	Parm (M)	GFLOPs
✓				2.37	6.7
✓	✓			2.38	6.8
✓		✓		5.32	12.6
✓			✓	2.38	6.7
✓	✓	✓		5.33	12.8
✓	✓		✓	2.39	6.8
✓		✓	✓	5.33	12.6
✓	✓	✓	✓	5.33	12.8

**Table 8:** Detection performance under synthetic infrared offset on LLVIP.

Offset	Baseline		+FARM	
	mAP_50_	mAP_50–95_	mAP_50_	mAP_50–95_
−16	0.9051	0.3626	0.9379	0.3883
−8	0.9380	0.5179	0.9642	0.5658
−4	0.9428	0.5786	0.9677	0.6369
−2	0.9448	0.5984	0.9681	0.6607
+2	0.9433	0.9675	0.9675	0.6660
+4	0.9442	0.9683	0.9683	0.6483
+8	0.9396	0.5429	0.9647	0.5844
+16	0.9191	0.3963	0.9429	0.4134

## Data Availability

The datasets utilized in the experiments are publicly accessible as follows: the KAIST dataset is available at https://github.com/SoonminHwang/rgbt-ped-detection/tree/master/data (accessed on 8 May 2026), the LLVIP dataset can be accessed at https://bupt-ai-cz.github.io/LLVIP/ (https://github.com/bupt-ai-cz/LLVIP or https://github.com/bupt-ai-cz/LLVIP/blob/main/Term%20of%20Use%20and%20License.md) (accessed on 8 May 2026), and the FLIR ADAS dataset is available at https://oem.flir.com/solutions/automotive/adas-dataset-form/#anchor1 (accessed on 8 May 2026).
